# Adoption of computerized tomography perfusion imaging in the diagnosis of acute cerebral infarct under optimized deconvolution algorithm

**DOI:** 10.12669/pjms.37.6-WIT.4884

**Published:** 2021

**Authors:** Bo Fang, Hongjiang Zhai

**Affiliations:** 1 Bo Fang, associate chief physician. Department of Neurology, Lu’an People’s Hospital Affiliated to Anhui Medical University, Lu’an, 237005, Anhui Province, China; 2 Hongjiang Zhai, chief physician. Department of Neurology, Lu’an People’s Hospital Affiliated to Anhui Medical University, Lu’an, 237005, Anhui Province, China

**Keywords:** Deconvolution optimization algorithm, Computerized Tomography Perfusion Imaging, Core infarct area, Acute cerebral infarct, Penumbra ischemic

## Abstract

**Objectives:**

To explore the significance of the hemodynamic parameters of Computerized Tomography Perfusion Imaging (CTPI) under the deconvolution optimization algorithm for the diagnosis and treatment of patients with acute cerebral infarct (ACI).

**Methods:**

A hundred and ten patients with ACI from December 2018 to September 2019 were selected for research, and CTPI was performed before and after Edaravone injection treatment. Then, the CTPI deconvolution algorithm based on the weighted adaptive (WA) total variation (TV) (WA-TV) optimization was constructed, which was compared with tensor total variation (TTV) and Motion-adaptive sparse parity (MASP). Brain Perfusion 4.0 was applied to obtain the relative time to peak (rTTP), the relative transit time of mean (rMTT), relative cerebral blood volume (rCBV), and relative cerebral blood flow (rCBF) of the core infarction area (CIA) and penumbra ischemic (PI).

**Results:**

In four parameters of rTTP, rMTT, rCBV, and CBF, the peak signal to noise ratio (PSNR) of the WA-TV algorithm was higher than the MSAP and TTV algorithms, while the Mean Square Error (MSE) and Mean Absolute Error (MAE) were lower than MSAP and TTV algorithms (P<0.05); the parameters of rCBV (71.56±9.87), rCBF (43.17±7.06) of the CIA before treatment were higher than PI (23.66±7.22; 18.37±3.99), rMTT (124.83±9.73) and rTTP (122.57±7.41) were lower than the PI (183.17±10.16); 150.74±9.74) (P<0.05). After treatment, the rCBV and rCBF of PI were higher than before treatment, and rMTT and rTTP were lower than before treatment (P<0.05), and there was no obvious difference in rCBV, rCBF, rMTT, and rTTP before and after treatment in the CIA (P>0.05).

**Conclusion:**

Compared with TTV and MASP, the WA-TV algorithm performs better in noise reduction and artifact reduction. The CTPI parameters of rCBV, rCBF, rMTT, and rTTP are all important indications for the diagnosis of PI and ACI.

## INTRODUCTION

ACI may occur if abnormal objects (solid, liquid, gas) enter the cerebral arteries or the neck arteries, block blood flow, and soften or even necrotize the brain tissues.^1^,^2^ The current clinical treatments for ACI include general treatment, thrombolytic therapy, and drug therapy. The general treatment nurses the patient’s skin, mouth, respiratory tract, and urine. Thrombolytic therapy adopts recombinant tissue-type plasminogen activator to treat patients with intravenous thrombolysis. Medications include mannitol, heparin, and Edaravone.^3^-^5^ CT can check the lesions of patients with ACI, with low cost, fast examination, and non-invasiveness. CTPI can check the brain with contrast agent injection^4^-^7^, providing help for the clinical development of personalized thrombolytic therapy. Deconvolution can calculate various myocardial perfusion hemodynamic parameters, including myocardial blood flow and myocardial blood volume, to assess myocardial ischemia.^8^-^10^

Hence, the CTPI deconvolution algorithm based on the WA-TV optimization was constructed to compare with the TTV and MASP algorithms. CTPI was performed before and after the Edaravone injection treatment on 110 cases with ACI, and the clinical value of CTPI was evaluated through comparison with the parameters of rTTP, rMTT, rCBV, and rCBF of the CIA and the PI.

## METHODS

One hundred ten patients with ACI admitted to the hospital from December 2018 to September 2019 were selected and performed CTPI before and after Edaravone injection treatment. The study was approved by the medical ethics committee of the hospital, and the patients and their families understood the study and signed an informed consent form.

### Inclusion criteria

I. Patients with the first onset; II. Patients with complete clinical data; III. Patients who have not received thrombolytic therapy; IV. Patients with non-responsible low-density lesions screened by CT scan; V. Patients with clear onset time.

### Exclusion criteria

I. Patients younger than 18 years; II. Patients who were unconscious and unable to cooperate with the examination; III. Patients with a history of epilepsy; IV. Patients who were allergic to iodine-containing contrast media; V. Those who had received anticoagulant treatment.

A 128-slice spiral CT scanner of General Electric (GE) can examine patients. The contrast agent was Iopromide Injection 300 (Iodine Concentration 300mg/mL) of Bayer Pharmaceuticals. Here, a high-pressure syringe was adopted to inject patients with 50 mL of Iopromide into the elbow vein at a rate of 5 mL/s, and the patients are scanned from head to foot. A total of 20 cycles are scanned to obtain a time-dose-curve (TDC). The scanning parameters were tube voltage 125kv, tube current 110mA, layer thickness 6mm, matrix 512×512, and field of view 240×240mm. After the CT scan, the perfusion image was sent to the Brain Perfusion 4.0 perfusion analysis software for the perfusion imaging parameters, namely, CBV, CBF, mean transit time (MTT), and time to peak (TTP). Then, according to the size of the CIA and the PI zone, the relative ratio of the affected side and the uninfected side of the abnormal perfusion area was obtained by mirror line symmetry, including rTTP, rMTT, rCBV, and rCBF.

Construction of deconvolution algorithm based on WA-TV optimization. First, the tracer dilution theory was introduced to construct a myocardial CT perfusion convolution model, that is, the arterial input function was used to convolve the blood flow scale function to obtain the tissue contrast agent concentration, the arterial input function was set as B, and the blood flow scaling function was set as G. Then, the convolutional discrete matrix of the region of interest of the entire organization can be expressed as follows.


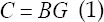


In which, *C* was the concentration of tissue contrast agent, *C=[c_1_,...,c_N_*] , *G=[g_1_,...,g_N_*], *N* was the number of voxels in the region of interest. Then, the regularization method can obtain a stable and accurate blood flow scale function, as shown in equation (2).


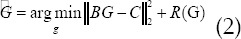


Equation (2) was the myocardial CT perfusion convolution model, *R(G)* represented the regularization term, and 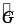 represented the blood flow scaling function. Using regularization to reduce the noise of CT perfusion images was a commonly used method, but the traditional TV has obvious shortcomings, which can cause image texture results to be lost due to step artifacts. To solve this problem, the WA-TV regularization method was introduced, and it performed adaptive adjustments according to the local image intensity to ensure the completeness of the image edge detail information, which was set as WA-TV. The objective function was as follows.


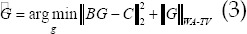



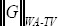
 represented the prior term of WA-TV regularization, which can be defined as follows.


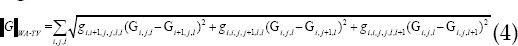



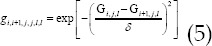



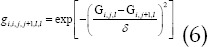



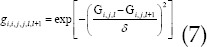


*δ* represented the scale factor for adjusting the iteration intensity, which was sensitive to changes in local voxel intensity. Then, the Iterative Shrinkage-Thresholding Method was introduced to optimize the WA-TV regularization prior terms, which can be expressed as follows.


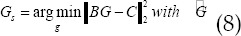



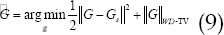


If *G_S_* was the solution of the nth iteration, then the equation below was obtained.


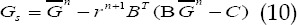


In which, 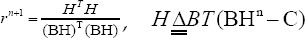. The above was the deconvolution algorithm based on WA-TV optimization. Evaluation indicators of the algorithm proposed. PSNR, MSE, and MAE can evaluate the performance of the WA-TV algorithm constructed, and TTV and MASP were introduced for comparison.


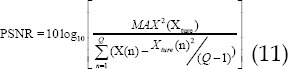



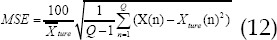



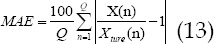


I*X* represented the pixel value of the area of interest, *X_ture_* represented the pixel value of the area of interest of the standard dose of contrast agent, *X̅_ture_* represented the average value of *X_ture_*, and *Q* represented the number of pixels.

### Statistical Analysis

SPSS19.0, mean ± standard deviation (x(-)± s), and the percentage (%) were adopted to process the count data. The comparison of PSNR, MSE, and MAE of WA-TV, MSAP, and TTV algorithms adopted t-test. The relative values of parameters in the PI and CIA before and after treatment were compared through variance. With *P*<0.05, the difference was statistically significant.

## RESULTS

Table-I showed that the proportion of male patients was slightly higher than that of females; patients older than 50 years was the highest; patients with body mass index of 27-29.9 was the highest; and patients with diabetes, hypertension, and hyperlipidemia were higher than those without them, and the proportions of patients with or without a smoking history didn’t differ much, neither did patients with or without coronary heart disease.

**Table-I:** Basic data of patients.

*Variable*	*Classification*	*Sample size (person)*	*Proportion (%)*
Gender	Male	59	53.64
	Female	51	46.36
Age (year)	<=50	11	10.00
	50-60	23	20.91
	60-70	44	40.00
	>=70	32	29.09
Body mass index	<23.9	18	16.21
	24-26.9	29	26.15
	27-29.9	44	40.28
	>=30	19	17.36
Diabetes	With	70	63.38
	Without	40	36.62
Smoking history	With	58	53.18
	Without	52	46.82
Hypertension	With	66	60.39
	Without	44	39.61
Hyperlipidemia	With	71	64.28
	Without	39	35.72
Coronary Heart Disease	With	59	54.02
	Without	51	45.98

**Fig.1:**
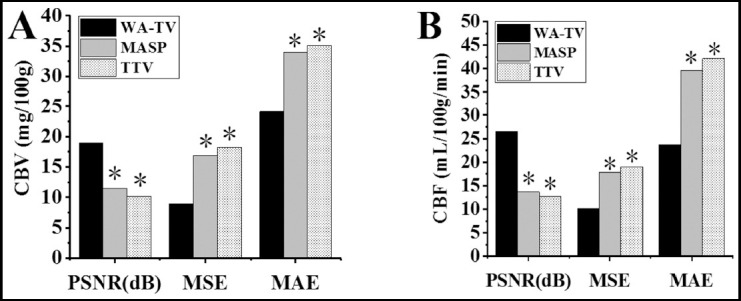
Performance of the three algorithms under different CT perfusion parameters. A was CBV; B was CBF; * indicated that the difference was obvious compared with the WA-TV (P<0.05).

**Fig.2:**
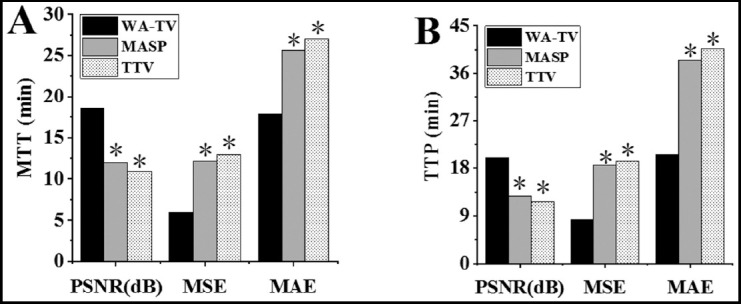
Performance of the three algorithms under different CT perfusion parameters. A was MTT; B was TTP. * indicated that the difference was obvious compared with the WA-TV (P<0.05).

Performance of the three algorithms under different CT perfusion parameters. In Fig.1 & 2, in the parameters of CBV, CBF, MTT, and TPP, the PSNR of the WA-TV algorithm was higher than that of the MSAP and TTV algorithms (*P*<0.05); the MSE and MAE of the WA-TV algorithm were lower than the MSAP and TTV algorithms (*P*<0.05).

**Fig.3:**
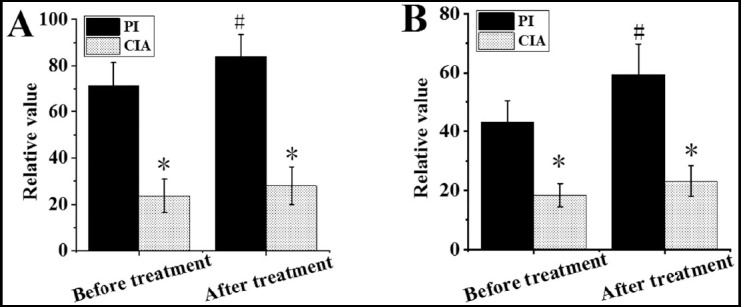
Comparison of relative values of PI and CIA parameters before and after treatment (rCBV and rCBF). A was rCBV; B was rCBF; * indicated that the difference was obvious compared with PI (P<0.05); # indicated that the difference was obvious compared with that before treatment (P<0.05).

**Fig.4:**
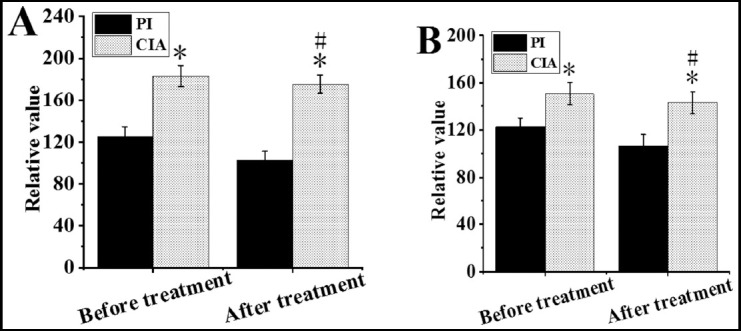
Comparison of relative values of PI and CIA parameters before and after treatment (rMTT and rTTP). A was the relative value of rMTT; B was the relative value of rTTP; * indicated that the difference was obvious compared with PI (P<0.05); # indicated that the difference was obvious compared with that before treatment (P<0.05).

Comparison of relative values of PI and CIA parameters before and after treatment. As shown in Fig.3 & 4, the rCBV and rCBF in PI after treatment were higher than before treatment, and rMTT and rTTP were lower than before treatment (*P*<0.05); the difference of rCBV, rCBF, rMTT, and rTTP before and after treatment in the CIA was not obvious (*P*>0.05).

## DISCUSSION

The results showed that the WA-TV algorithm proposed in this study had a higher PSNR in contrast to the TTV and MASP algorithms after the CTPI images were processed. The PSNR index is a quantitative index used to evaluate the effect of image reconstruction. The larger the PSNR value, the closer the resolution of the reconstructed image to the original image.^11^-^13^ Therefore, it suggested that the resolution of the CTPI image reconstructed by the WA-TV algorithm proposed in this study was higher, and the reconstruction result was significantly better than that of the TTV and MASP algorithms.

Subsequently, the CTPI images processed by the WA-TV algorithm was applied to compare and analyze the changes in the brain tissue structure of patients with ACI before and after treatment. The indicators used to evaluate the effect of the indicators included CBV, CBF, MTT, and TTP. CBV refers to the VOLUME of blood through a certain cross-section of cerebrovascular in a unit time, which can reflect the damage of brain tissue.^14^-^15^ CBF refers to the total amount of blood contained in cerebral blood vessels (including cerebral arteries, arterioles, and capillaries), and it has important guiding value for the clinical treatment of patients with acute cerebral infarction.^16^,^17^. MTT means the average time for blood to pass through the vasculature of a specific brain area.^18^,^19^ TTP refers to the time required for the contrast agent to reach the maximum concentration of the brain tissue.^20^ The results of this study revealed that CBV and CBF were much higher in patients with ACI before treatment, while MTT and TTP values were greatly lower. It showed that treatment can dramatically improve the perfusion state of ischemic brain tissue in patients with ACI, and reduce the damage to the brain tissue and nerve function of patients.

## CONCLUSION

Here, the CTPI deconvolution algorithm was constructed based on the WA-TV optimization, the performance was compared with TTV and MASP, and CTPI scanned patients with ACI. Consequently, compared with TTV and MASP, the WA-TV algorithm has better noise reduction and artifact reduction. The CTPI parameters of rCBV, rCBF, rMTT, and rTTP are all important indications for the diagnosis of PI and ACI.

### Limitations of the study

The sample size is small, it has not been compared with normal brain tissue. Later, an enlarged patient sample should be considered to further explore the effectiveness of the deconvolution algorithm. In short, the results provide a reference for the application of CTPI parameters based on the deconvolution algorithm in the diagnosis of ACI.

### Authors Contribution:

**BF:** Conceived the study, literature review, analysis of data and drafting the manuscript.

**HZ:** Takes the responsibility and is accountable for all aspects of the work in ensuring that questions related to the accuracy or integrity of any part of the work are appropriately investigated and resolved.
